# Comparative proteomics analysis reveals differentially accumulated proteins associated with male and female *A. chinensis* var. *chinensis* bud development

**DOI:** 10.1186/s12953-021-00176-w

**Published:** 2021-04-22

**Authors:** Yu Zhang, Yuexing Wang, Wanying Zhou, Shimao Zheng, Wenhui Zhang

**Affiliations:** 1 School of Biological Sciences and Engineering, Shaanxi University of Technology, Hanzhong, Shaanxi China; 2 College of Agronomy, Xinjiang Agricultural University, Urumqi, Xinjiang China; 3 Ankang Municipaty Agricultural Sciences Rese arch Institute, Ankang, Shaanxi China

**Keywords:** *A. chinensis* var. *chinensis*, Dioecious, Bud, Label-free quantification, Proteomics, Sex differentiation

## Abstract

**Background:**

Kiwifruit (*Actinidia chinensis* var. *Chinensis*) is abundant with vitamin C and is a rapidly developing crop in China, New Zealand, and other countries. It has been widely used as a raw material for food and kiwifruit wine. Among these, *A. chinensis* var. *chinensis* and *A. chinensis* var. *deliciosa* are the most valuable kiwifruit in production. Kiwifruit is a typical dioecious plant and its female and male plants have different economic values. Therefore, sex identification, especially at the seedling stage, has important implications for the scientific planning of its production and economic benefits. However, the kiwifruit sex regulation mechanism is very complex and molecular studies are in the initial stages. Currently, there is not a universal and effective sex identification method for *A. chinensis.*

**Methods:**

In this study, we used a label-free quantitative proteomics approach to investigate differentially accumulated proteins, including their presence/absence and significantly different levels of abundances during *A. chinensis* var. *chinensis* male and female flower bud development*.*

**Results:**

A total of 6485 proteins were identified, among which, 203 were identified in male buds, which were mainly associated with phenylalanine metabolism, tyrosine metabolism, and plant hormone signal transduction. In female buds, 241 were identified, which were mainly associated with the ErbB signaling pathway, growth hormone synthesis, secretion and action, and mRNA surveillance pathway. A total of 373 proteins were significantly differentially accumulated proteins (fold change > 2; *P* < 0.05), of which, 168 were upregulated and 205 were downregulated. Significant differences between proteins involved 13 signaling pathways, most of which were involved in flavonoid biosynthesis, phenylpropanoid biosynthesis, and starch and sucrose metabolism. Protein interaction analysis showed that enriched protein nodes included cell division cycle 5-like protein, 40S ribosomal protein S8, ribosomal protein, and 40S ribosomal protein like, which interact with 35, 25, 22, and 22 proteins, respectively.

**Conclusions:**

This study provide valuable information for cloning key genes that control sex traits and functionally analyze their roles, which lay a foundation to the development of molecular markers for male and female kiwifruit identification.

**Supplementary Information:**

The online version contains supplementary material available at 10.1186/s12953-021-00176-w.

## Introduction

*Actinidia chinensis* var. *chinensis* and *A. chinensis* var. *deliciosa* are the most valuable kiwifruit in production*.* As a typical dioecious plant, research has shown that using male plants as rootstocks has strong grafting advantages, but most rootstocks currently used in production consist of 1–2-year-old seedlings. Because there is not a good method for distinguishing male and female plants at the seedling stage, there are both female and male plants being used as rootstocks. Thus, there is inconsistent growth of fruit trees, which complicates the later stages of management and leads to inconsistent kiwifruit quality. Additionally, the relatively long juvenile kiwifruit phase lengthens its breeding time. Therefore, it is important to identify the gender of kiwifruit at the seedling stage. Since the 1980s, researchers have studied genetic markers between male and female plants, including morphological markers, flavonoid content [1], soluble sugar content [2], chlorophyll content [3], and protective enzyme activity [4]. However, these genetic markers are unstable due to their sensitivity to the environment and have not been used effectively in practical applications. Thus, the goal of this study was to resolve the sex-determining mechanism of kiwifruit, which lay a foundation to develop molecular markers to identify gender at the seedling stage.

The sex determination mechanism of dioecious plants is very complex. Sex chromosomes, sex genes, and transcription factors are the key genetic factors that affect the development of male and female individuals and sex organs. Sex determination and sex differentiation are two processes of male and female plant development, but the differentiation process and regulation mechanism remain unclear. Thus far, only a few reports exist on kiwifruit sex development. In *A. chinensis* var. *chinensis*, the literature suggests that chromosome 25 may be the sex-chromosome [5] and a male related gene, friendly boy (*Frby*), has been identified [6]. *Frby* was cloned and the sequence was mapped to chromosome 8 in female *A. chinensis* var. *Chinensis* [7], indicating that the homologous sequences on the X chromosome that correspond with *Frby* vary considerably. Clearly, the sex differentiation mechanism in kiwifruit is very complex and requires further investigation.

Recently, the sex development mechanism has been studied in dioecious and monoecious plants via proteomics technology on the differential expression of genes/proteins, and provide a theoretical basis for the mechanism of flower bud development in different sexed plants. Sex-related differential proteins of *Pistacia chinensis* were identified using the proteomic method and it was speculated that phosphoglycerate kinase and temperature-induced lipid delivery proteins may be molecular markers related to gender differences in *Pistacia chinensis* [8]. Moreover, in the dioecious and monoecious plants of *Pistacia chinensis*, differentially expressed proteins were detectable during antioxidant stress, ribosome activity, and photosynthesis [9]. According to research on differentially expressed genes in male and female flower buds of *Ginkgo biloba* (Xiaowen), it was speculated that some female flower buds are located in the female specific region of the W chromosome and homologous gene sequences are present on the Z chromosome [10].

Until now, kiwifruit sex differentiation has rarely been studied. In order to explore the sex differentiation mechanism of *A. chinensis* var. *chinensis*, this study employed the labor-free quantitative proteomics method to compare and analyze related differential proteins between male and female flower buds of *A. chinensis* var. *chinensis* for the first time. The purpose of this study was to provide valuable information for cloning key genes that control sex traits and functionally analyze their roles, as well as lay a foundation to develop molecular markers to identify gender at the seedling stage.

## Materials

### Proteins extraction and peptide digestions

The male and female flower buds (about 2 cm in diameter) were collected from the kiwifruit experimental farm during the 2019 growing season in Ankang Municipaty Agricultural Sciences Research Institute, China, (E:108°47′47″, N:32°43′56″) on April 25th, with three biological replicates for each sample, and sample was ground individually in liquid nitrogen.

For cell lysis, cell pellets were suspended on ice in 200 μL SDT (4%SDS, 100 mM Tris-HCl, 1 mM DTT, pH 7.6) lysis buffer. The supernatant were collected and quantified with a BCA Protein Assay Kit (Bio-Rad, USA).

Digestion of protein was performed according to the FASP (filter-aided sample preparation) procedure described by Wisniewski J.R [11].. The peptide of each sample was desalted on C18 Cartridges (Empore™ SPE Cartridges C18 (standard density), bed I.D. 7 mm, volume 3 ml, Sigma), then concentrated by vacuum centrifugation and reconstituted in 40 μl of 0.1% (v/v) formic acid. The peptide content was estimated by UV light spectral density at 280 nm.

### Liquid chromatography (LC) - electrospray ionization (ESI) tandem MS (MS/MS) data acquisition

Each sample was separated by HPLC with an Easy-nLC system (Thermo Fisher Scientific), which was coupled to a Q-Exactive Mass Spectrometer (Thermo Scientific). The samples was loaded onto the column (Thermo Scientific Acclaim PepMap100, 100 μm*2 cm, nanoViper C18) in buffer A (0.1% (v/v) formic acid), and separated by analytical column (Thermo Scientific EASY column, 10 cm long, 75 μm inner diameter, 3 μm resin, C18-A2) with a linear gradient of buffer B (84% (v/v) acetonitrile and 0.1%(v/v) formic acid)at a flow rate of 300 nl/min, the column was re-equilibrated with 95% buffer A.

MS data was acquired using a data-dependent top10 method dynamically choosing the most abundant precursor ions from the survey scan (300–1800 m/z). Determination of the target value is based on predictive Automatic Gain Control (pAGC). Target value for the full scan MS spectra was 3 × 106 charges in the 300–1800 m/z range with a maximum IT(injection time) of 50 ms and a resolution of 70,000 at m/z 200, dynamic exclusion duration was 60s. The mass charge ratio of polypeptide and polypeptide fragment was determined according to the following method set: 20 fragments (MS2 scan) were collected after each full scan, and the MS2 activation type was HCD, isolation window was 2 m /z, the resolution of secondary mass spectrometry was 17,500 at 200 m / z, the Normalized Collision Energy was 30 eV and the underfill ratio, which specifies the minimum percentage of the target value likely to be reached at maximum fill time, was defined as 0.1%.

### Protein identification and quantification analysis

Raw files were processed with MaxQuant (v 1.5.3.17) using the standard settings against a *Actinidia chinensis* protein database (uniprot_Actinidia_chinensis_33232.fasta, 76,417 total entries, downloaded 2014/12/12). An initial search was set at a precursor mass window of 6 ppm. The search followed an enzymatic cleavage rule of Trypsin/P and allowed maximal two missed cleavage sites and a mass tolerance of 20 ppm for fragment ions. The cutoff of global false discovery rate (FDR) was set to 1% for protein and peptide identifications. Protein aboundance was calculated on the basis of the normalized spectral protein intensity (LFQ intensity) [12].The differentially accumulated proteins, including proteins of presence or absence abundant (with two or more null values in one group samples), and proteins were defined as regulated with at least 2 fold changes and a *p*-value ≤0.05 between male flower buds and female flower buds.

### Identification of differentially expressed proteins

The differentially expressed proteins, including proteins of presence or absence expressed (with two or more null values in one group samples), and significant differentially expressed proteins (up-regulated more than 2 fold or down regulated less than 0.5 and *p*-value< 0.05) were screened by UniProt database (https://www.uniprot.org/). The differentially expressed proteins were analyzed; follow-up bioinformatics.

### Bioinformatic analysis

#### Gene ontology (GO) term

Bast2Go (HTTPS:0/www.blast2go.com/) software was employed to annotate the GO term of all the proteins identified in this study. The process of GO annotation includes Blast, Mapping, Annotation, and Annotation Augmentation was conducted in InterProScan (https://www.ebi.ac.uk/interpro/search/sequence/).

#### Kyoto encyclopedia of genes and genomes (KEGG) pathway

KAAS (KEGG automatic annotation server) was employed to annotate the KEGG pathway.

#### GO and KEGG pathway enrichment analysis

The enrichment analysis of GO term or KEGG pathway was performed by Fisher’s exact test.

### Protein clustering analysis

Hierarchical clustering method was used to cluster the differentially accumulated proteins in the male and female flower buds, and the results were displayed in the heat map.

### Protein-protein interactions (PPIs) analysis

To investigate how these significantly differentially accumulated proteins (A total of 373 proteins) are functionally associated with each other, PPI analysis was conducted by using the String (https://string-db.org/), the corresponding results from the String database were downloaded in STV format, then visualized and edit by Cytoscape software (http://www.cytoscape.org/, v3.7.1), as well as further analyze the degree of each targeted protein within the PPI network, which estimate its corresponding significance.

## Results

### Protein identification

**Fig. 1:**
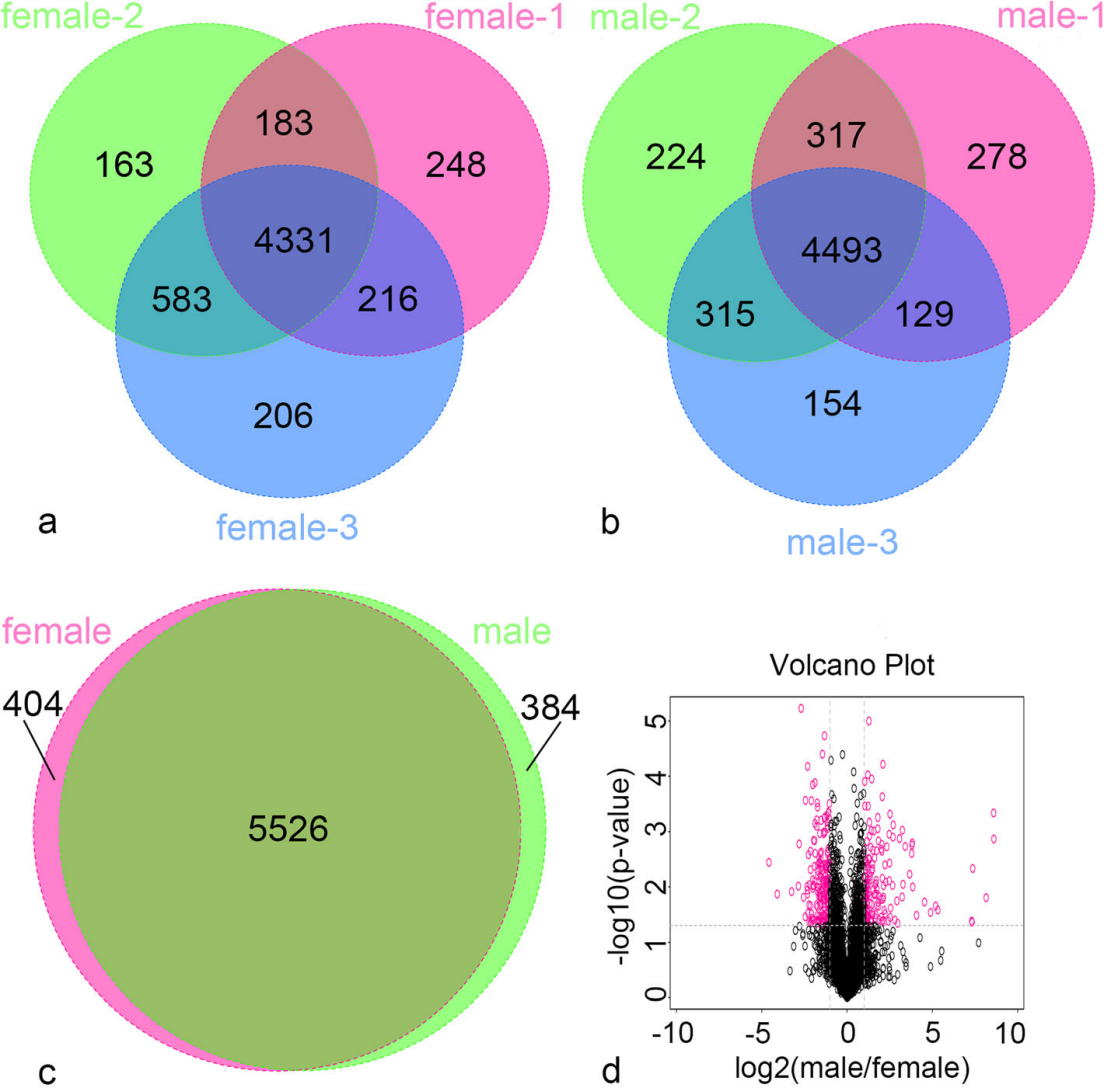
Differentially accumulated proteins (DAPs) analysis: **a** Venn diagram of protein identified in female group. **b** Venn diagram of protein identified in male group. **c** Venn diagram of DAPs in multiple pairwise comparison. **d** The volcano map of DAPs analyzed in male vs female. Note: In the Fig. 1d, the red dot is the protein with significant differentially accumulated proteins, and the black dot is the protein without difference change

A total of 6485 proteins were identified. Among these, 404 proteins were found in female plants, 384 in male plants, and 5526 identical proteins were found both males and females (Fig. 1c).

### Differential accumulated proteins screening

The number of differentially accumulated proteins in each comparisons are shown in Table 1. Consistent presence/absence abundant profile means the differential proteins with two or more non null values in one group of samples and null values in the other group. Significantly changing in abundance means fold change between the two groups of samples and *p*-value obtained by T test, were used to draw the volcano map (Fig. 1d).

**Table 1:** Data statistics of protein quantitative test

Comparisons	Consistent presence/absence abundant profile		Significantly changing in abundance	
	Increased	Decreased	Increased	Decreased
**Male vs Female**	203	241	168	205

### Cluster analysis of differentially accumulated proteins

**Fig. 2:**
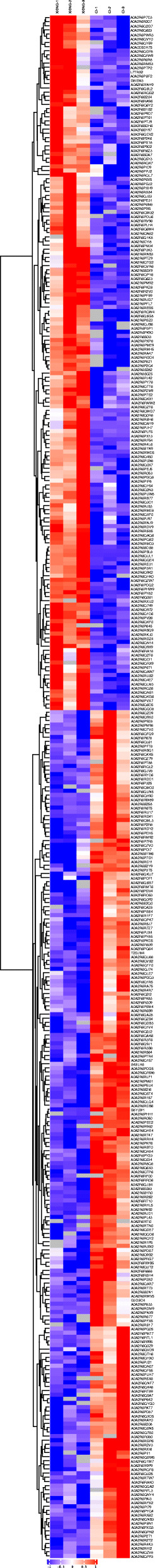
Heatmap of the abundances levels of the 373 proteins in male vs female buds using the laber-free quantitative protein test. Note: In Fig. 2, each row represents a protein, each column represents a group of samples, in which red represents a significant up-regulated protein, blue represents a significant down-regulated protein, and gray represents no protein quantitative information

Using the hierarchical cluster to cluster the differentially accumulated proteins in the comparison group, the genes usually classified as one group have practical relationship in some biological processes, or in some metabolism and signal pathway. In this study, proteins with similar accumulated patterns were clustered together and displayed in the form of heat map by differentially accumulated proteins (Fig. 2). It were identified 373 proteins that were significantly differentially accumulated between male and female buds. These differentially accumulated proteins were mainly divided into two clusters, of the 205 proteins which were up-regulated in female and down-regulated in male, 168 which were up-regulated in male and down-regulated in female. It can be seen that there is a significant difference in protein expression between male and female flower buds of *A. chinensis*, indicating that different proteins have different functions and ways of action between the development and differentiation of male and female flower buds.

### GO enrichment analysis of differentially accumulated proteins

GO functional enrichment analysis showed that 1257 terms were annotated including 710 terms in the biological process (BP), 365 terms in the molecular function (MF) and 185 terms in the cellular component (CC).

### GO enrichment analysis of proteins of presence or absence

**Fig. 3:**
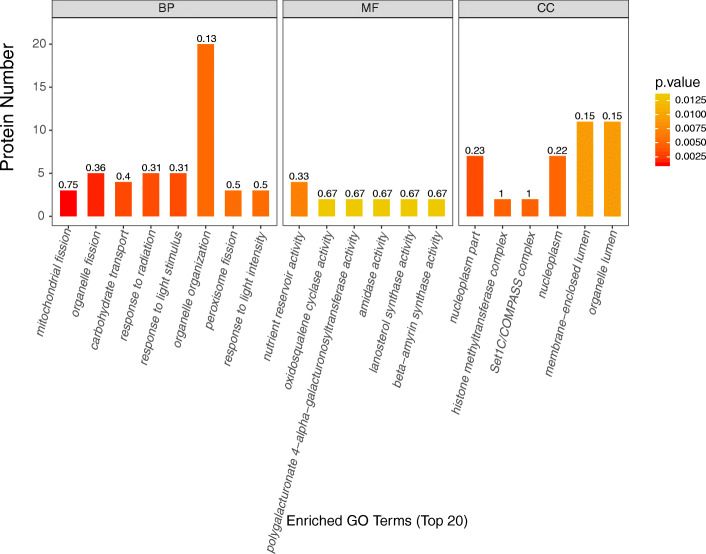
The top 20 GO enrichment of proteins of presence/absence in male vs female buds

The top 20 functional enrichment proteins were analyzed (Fig. 3). For biological processes, mitochondrial fission is a representative GO term, followed by organelle fission. Among the molecular functions, the nutrient reservoir activity is highly representative. For cell components, nuclear part, histone methyltransferase complex, set1c/Compass complex are highly representative.

### GO enrichment analysis of significant differentially accumulated proteins

**Fig. 4:**
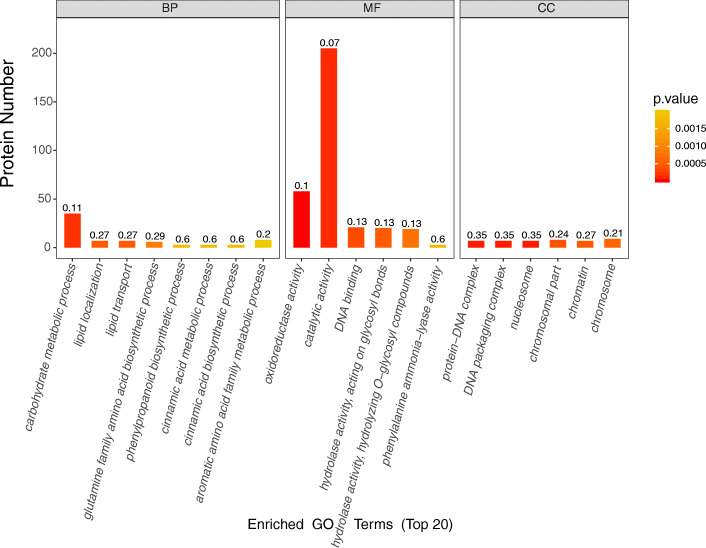
The top 20 GO enrichment of significant differentially accumulated proteins in male vs female buds

For biological processes, carbohydrate metabolic process is a representative GO term, followed by lipid localization and lipid transport. Among the molecular functions, the proteins with redox activity and catalytic activity were highly representative. For cell components, protein DNA complex and DNA packaging complex (DNA packaging) were highly representative. Complex and nucleosome were also highly representative (Fig. 4).

### KEGG enrichment analysis of differentially accumulated proteins

#### KEGG enrichment analysis of proteins of presence or absence

**Fig. 5:**
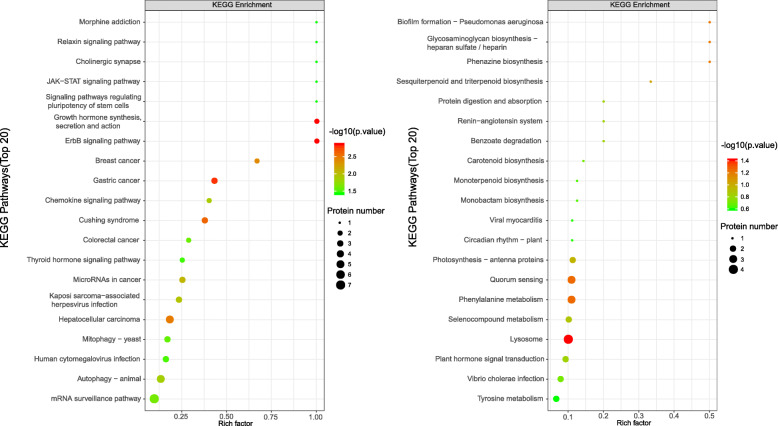
The top 20 KEGG pathway enrichment of differentially accumulated proteins in male vs female buds; a. Proteins accumulated only in female buds. b. Proteins accumulated only in male buds

KEGG enrichment pathway of proteins only in female buds or in male buds was showed in Fig. 5, among which ErbB signaling pathway, growth hormone synthesis, secretion and action, mRNA surveillance pathway are occured mainly in female plants (Fig. 5a), and phenylalanine metabolism, tyrosine metabolism and plant hormone signal transduction are occured mainly in male plants (Fig. 5b). Phenylalanine metabolism is one of the most important pathways of plant secondary metabolism [13], which includes mainly two metabolic pathways, namely phenylpropanoid pathway and flavonoid biosynthesis pathway [14–16].

**Fig. 6:**
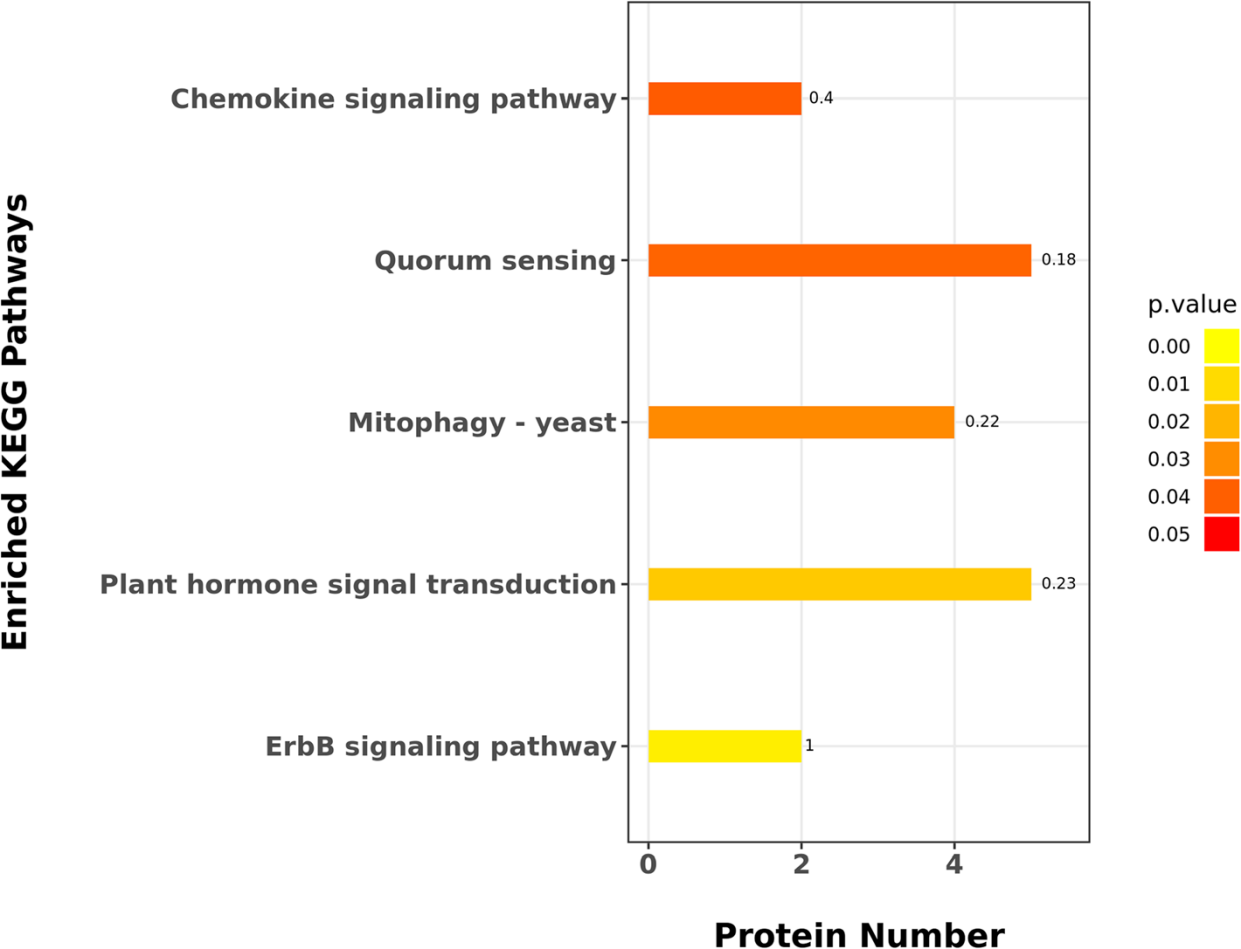
The KEGG pathway enrichment of identified proteins on presence/absence in male vs female buds

KEGG enrichment pathway of all proteins that contain expressed only in female and in male buds was showed in Fig. 6, five metabolic pathways have been found by analyzing whether there are differential proteins enriched in KEGG pathway, in order of *P*-value from small to large, including ErbB signaling pathway, plant hormone signal transduction and so on.

Among all pathways, the plant hormone signal transduction was the most important, in which, five proteins were identified, including jasmonic acid-amido synthetase and auxin transporter-like protein, which were expressed only in male buds, and three proteins that were expressed only in female buds (Table 2). jasmonic acid-amido synthetase is an enzyme in the jasmonates metabolism pathway [17]. Jasmonate is an important class of lipid-derived plant hormone that regulates multiple aspects of plant growth and development, as well as plays key roles in stamen development [18–23]. jasmonic acid-amido synthetase (Jar1) is a regulatory gene that regulates the metabolism of jasmonic acid (JA) to jasmonic acid-isoleucine conjugate (Ja-ile). Wang Jing et al. [24] showed that the reproductive activities of the root knot nematode in a JA deletion mutant and JA-ile deletion mutant (aos1, opr3, Jar1) were significantly lower than the wild type. Exogenous Ja-ile restored the reproductive capacity of root knot nematodes in Jar1 roots to the same level as wild type roots. Dai Liangying et al. [15] analyzed Jasmonate functioning and its biosynthesis and metabolic pathway, and found that some jasmine are involved in growth and pollen development, while some are resistant to insects, disease, and stress through the participation of Jar1. It was also proved that auxin regulates stamen development [25] and there were no stamen or abnormal anther morphology as the germination rate of anthers decreased in an auxin transporter protein mutant [26]. Once the auxin transporter is destroyed, it affects stamen development, which thereby affects pollination and fertilization, resulting in reduced fruit yield and quality.

**Table 2:** Enriched pathway information of plant hormone signal transduction

Protein ID	Gene name	Protein description	Chromosome	Gender
A0A2R6Q7S6	CEY00_Acc19795	Jasmonic acid-amido synthetase	LG18	Male
A0A2R6R6W9	CEY00_Acc10793	Auxin transporter-like protein	LG9	Male
A0A2R6PMH5	CEY00_Acc28148	Abscisic acid receptor like	LG24	Female
A0A2R6PZF3	CEY00_Acc23499	Serine/threonine-protein kinase	LG21	Female
A0A2R6RBH6	CEY00_Acc09459	Histidine-containing phosphotransfer protein	LG18	Female

### KEGG enrichment analysis of significant differentially accumulated proteins

**Fig. 7:**
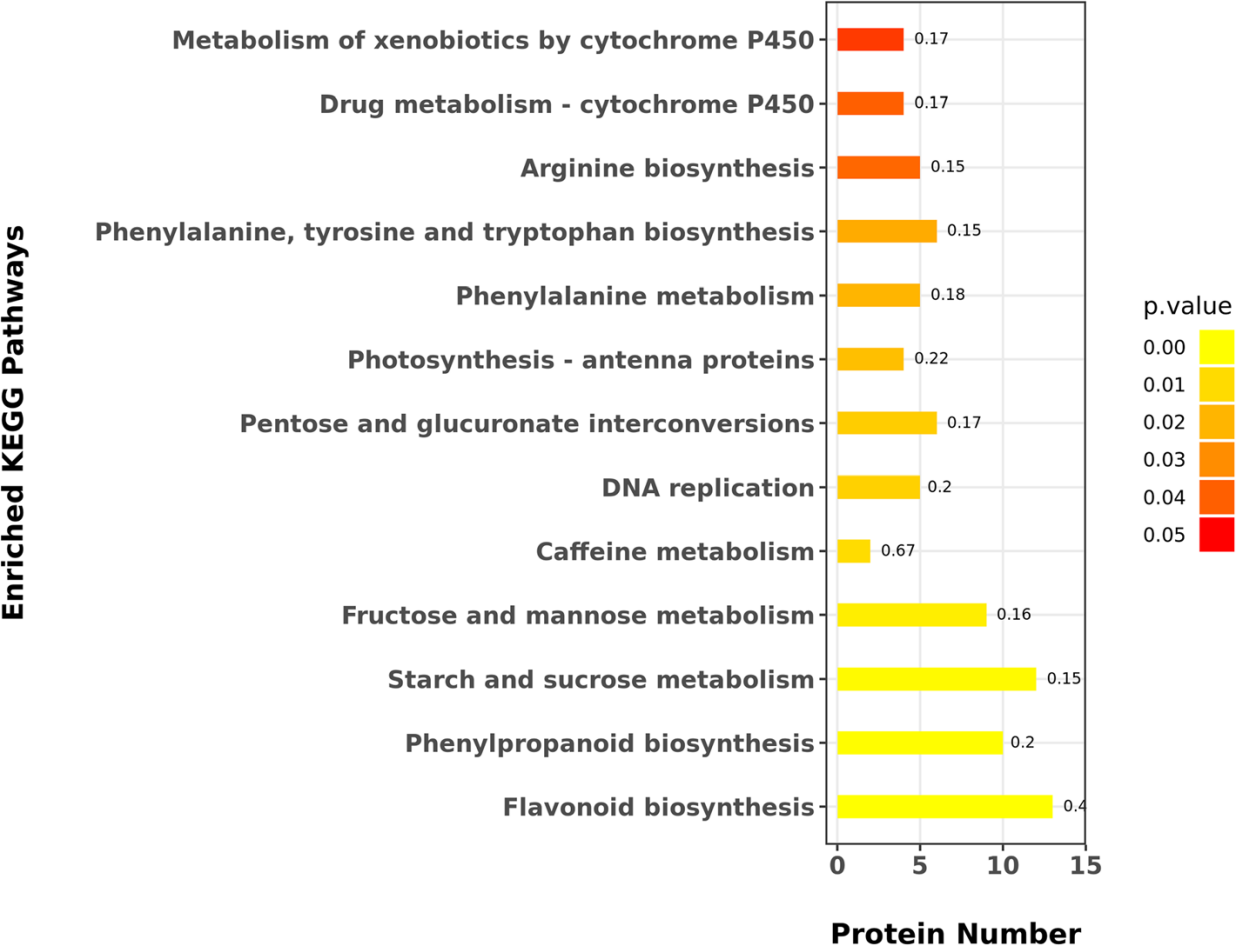
The KEGG pathway enrichment of significantly differential accumulated proteins in male vs female buds

In the KEGG pathway (Fig. 7), the first three pathways were flavonoid biosynthesis, arginine biosynthesis, and starch and sucrose metabolism. Flavonoid serves many functions, among which, it can attract pollination and regulate seed germination through flower and fruit pigmentation. Previous studies showed that a *Petunia hybrida* mutant exhibited pollen sterility, while wild-type stigma possess a certain substance that restores pollen fertility. The analysis found that this substance is a flavonoid compound that can promote pollen tube growth and pollen function [27]. Arginine serves a variety of functions that promotes cell division, seed germination, flower bud differentiation, root growth, and development. In flower bud differentiation, arginine synthesizes polyamine to promote bud and pollen germination and increases the fruit setting rate [28]. During arginine metabolism, putrescine is synthesized first, then polyamine is synthesized.

### Network analysis of protein-protein interaction (PPI)

**Fig. 8:**
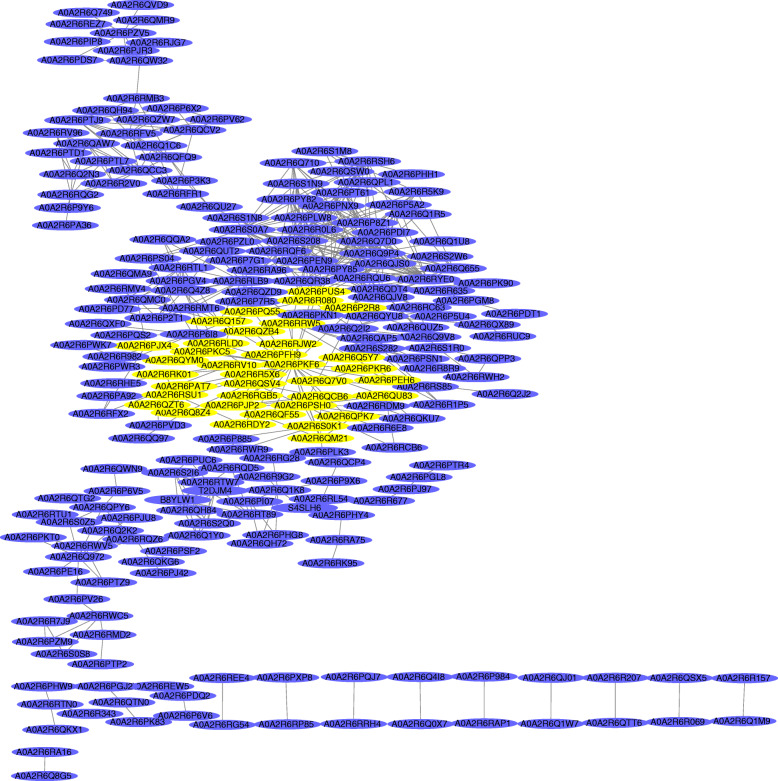
PPI networks of proteins from differentially identified proteins in the bud

Protein-protein interactions (PPIs) engage in dynamic biological processes and play important roles in these diverse processes, and are essential for understanding life at the system-level. Results revealed 243 nodes (proteins) and 542 edges (interactions), indicating a highly profound network of sex differentiation of *A. chinensis* var. *chinensis* buds (Fig. 8). Aoa2r6pkf6, a cell division cycle 5-like protein (Cdc5L), was a node protein that interacted with 35 proteins (yellow); the proteins that interacted with Cdc5L were mainly related to ribosome formation. The expression level of Cdc5L in female buds was 2.22-fold higher than male buds. A previous study found that Cdc5L is a regulator of mitotic progression as a pre-mRNA splicing factor. The deletion of endogenous Cdc5L decreases cell viability via dramatic mitotic arrest [29], which has not been reported in plants, but has been widely studied in animals, especially in the proliferation of cancer cells [30]. Cdc5L is also essential for porcine oocyte maturation [31]. Cdc5L could be a potential molecular marker in neuroblastoma [32] and may play an important role in mitosis and cell activity during female flower bud formation.

## Discussion

In production, dioecious plants are utilized differently based on sex. The reproductive function of female plants is higher than male plants, but the growth potential of female plants is lower than male plants [33]. The different evolutionary directions of male and female plants determine their growth patterns [34], and plants exhibit different stress responses to environmental stress based on sex [35]. Studies on kiwifruit production using male plants as rootstocks showed that they have a strong advantage. Significant differences were detected mainly between molecular functional proteins, of which, most were involved in catalytic activities. In the KEGG enrichment analysis, the plant hormone signaling pathway was mainly involved in the male and female plants comparison, which indicated that the signaling pathways of male and female flower bud development and differentiation were different, while the pathway of significantly different proteins mainly concentrated on flavor biosynthesis, phylopanoid biosynthesis, and sucrose metabolism. Studies on the content differences of key substances in the significant pathways between male and female plants and sex changes induced by these key substances in kiwifruit have been reported. Among them, the effects of hormones and polyamines on sex differentiation in horticultural plants are more frequently reported [36]. Zheng Hanyu et al. [1] reported that the flavonoid content in female kiwifruit leaves was higher than male leaves. Xu Linyue et al. [37] found that the content of putrescine and spermidine in male leaves was significantly higher than female leaves, and the spermine content in female leaves was significantly higher than male leaves. Studies have also shown that exogenous putrescine and spermidine increase the number of plant flower buds [28]. Hale et al. [38] used genotyping-by-sequencing (GBS) technology to locate a sex-linked marker, ac36306, in the natural population of Chinese gooseberry in the United States. After analysis, it was found that the marker was closely linked to arginine decarboxylase, a key enzyme in the polyamine biosynthesis pathway from arginine to putrescine. Zhu Daye et al. [2] found that the soluble sugar content in male leaves was significantly higher than female leaves. Understanding which genes and metabolic pathways are involved in the sex developmental process of Chinese kiwifruit is important for developing androgynous markers suitable for different natural populations of kiwifruit varieties.

DNA molecular markers have also been reported for kiwifruit [5, 38–40]. Accordingly, we identified male and female plants in natural populations of *A. chinensis* var. *chinensis* and *A. chinensis* var. *deliciosa* at the seedling stage*,* however, none of the aforementioned DNA markers were 100% accurate. This may be due to the fact that most of the mapping populations are specific populations (most were F2) and the DNA markers are only suitable for specific materials. Therefore, it may be that these DNA markers do not co-segregate with sex-determining genes during meiosis; the exchange between markers and sex-determining genes will occur, which will lead to false positives or negatives.

By combining the protein interaction network analysis and pathway annotation results, we obtained a more comprehensive and systematic view of male and female development at the molecular level, which will aid further research and mining for molecular mechanisms. The protein interaction network analysis showed that most proteins interacted with Cdc5L, which promotes cell activities. According to the GO annotations, the molecular function of the protein family is DNA binding and the biological process is cellular response to fibroblast growth factor stimulus (FGF). The genes involved in microspore development and pollen formation in *Arabidopsis* are cellulose loss deposition [41]. Cdc5L stimulates fibroblast growth and may participate in pollen formation. Through the GO, KEGG, and PPI analyses, the differences between male and female plants ultimately resulted in sex differentiation.

The sex determination mechanism of dioecious plants is very complex. Sex chromosomes, sex genes, and transcription factors are the key genetic factors that affect the development of male and female individuals or organs. Sex determination and sex differentiation are two processes of male and female plant development. Plant sex can be determined only at the reproductive growth stage. However, the differentiation process and regulation mechanism remain unclear, and research on kiwifruit sex development is limited. DNA molecular markers, which are used to distinguish male and female *A. chinensis* var. *chinensis*, were all located at different positions on chromosome 25 [5]. Further analysis indicated that most genes on chromosome 25 are associated with the metabolic pathways of plant hormone signal transduction. Thus, it was inferred that chromosome 25 may play a key role as a sex chromosome in *A. chinensis* var. *chinensis* sex development and differentiation. Additionally, among the differentially accumulated proteins that were identified, we found that the number of stress resistance proteins in male plants was greater than in female plants, indicating that male plants are more stress resistant. This finding was consistent with the results of previous studies, which found that male plants were more salt and drought tolerant than female plants [42]. Our findings are also consistent with practical applications, wherein male plants used as rootstocks possess higher growth dominance in production.

## Conclusion

Taken together, This study employed the labor-free quantitative proteomics method to compare and analyze related differentially accumulated proteins between male and female flower buds of *A. chinensis var. chinensis* for the first time, which provide valuable information for cloning key genes that control sex traits and functionally analyze their roles, and lay a foundation to the development of molecular markers for male and female kiwifruit identification. Combined with genome-wide association study (not published), in the future, we will focus on several candidate genes/proteins that occured in KEGG enrichment analysis, such as jasmonic acid-amido synthetase, auxin transporter-like protein and so on.

## Supplementary Information

**Additional file 1.**

**Additional file 2.**

**Additional file 3.**

## Data Availability

The datasets used and analyzed during the current study are available from the corresponding author upon reasonable request.

## Abbreviations

Frby: Friendly boy
GO: Gene Ontology
KEGG: Kyoto Encyclopedia of Genes and Genomes
PPIs: Protein-protein interactions
DAPs: Differentially accumulated proteins
BP: Biological process
MF: Molecular function
CC: Cellular component
Cdc5L: Cell division cycle 5-like protein Chronic wrap restraint stress
GBS: Genotyping-by-sequencing
FGF: Fibroblast growth factor stimulus
